# State and Trait Anxiety Share Common Network Topological Mechanisms of Human Brain

**DOI:** 10.3389/fninf.2022.859309

**Published:** 2022-06-23

**Authors:** Yubin Li, Lili Jiang

**Affiliations:** ^1^CAS Key Laboratory of Behavioral Science, Institute of Psychology, Beijing, China; ^2^Department of Psychology, University of Chinese Academy of Sciences, Beijing, China

**Keywords:** MRI, human brain, anxiety, centrality, efficiency

## Abstract

Anxiety is a future-oriented unpleasant and negative mental state induced by distant and potential threats. It could be subdivided into momentary state anxiety and stable trait anxiety, which play a complex and combined role in our mental and physical health. However, no studies have systematically investigated whether these two different dimensions of anxiety share a common or distinct topological mechanism of human brain network. In this study, we used macroscale human brain morphological similarity network and functional connectivity network as well as their spatial and temporal variations to explore the topological properties of state and trait anxiety. Our results showed that state and trait anxiety were both negatively correlated with the coefficient of variation of nodal efficiency in the left frontal eyes field of volume network; state and trait anxiety were both positively correlated with the median and mode of pagerank centrality distribution in the right insula for both static and dynamic functional networks. In summary, our study confirmed that state and trait anxiety shared common human brain network topological mechanisms in the insula and the frontal eyes field, which were involved in preliminary cognitive processing stage of anxiety. Our study also demonstrated that the common brain network topological mechanisms had high spatiotemporal robustness and would enhance our understanding of human brain temporal and spatial organization.

## Introduction

Anxiety is a future-oriented emotional state activated by potential and distant threats (Eysenck et al., 2007; Calhoon and Tye, 2015). It is unpleasant, negative, out of proportion to the threat and can be characterized by aversive and unpleasant avoidance behaviors (Spielberger et al., 1983; Grachev and Apkarian, 2000; Eysenck et al., 2007; Modi et al., 2015). Anxiety is different from fear concerning its association with the anticipation of uncertain threats and can be more easily triggered by distant and unpredictable threats (Geng et al., 2018). Spielberger (1966) suggested that anxiety could be conceptualized into multiple dimensions by distinguishing trait anxiety from state anxiety. Specifically, trait anxiety, as a personality dimension, can be defined as an individual's predisposition to worry about future threating events (Spielberger et al., 1983), and individuals with high trait anxiety have weakened image processing on the conscious level, stronger induced sensitivity, and a tendency to overprocess relationships (Yin et al., 2016). While state anxiety reflects a temporary, transient, and subjective emotion characterized by physiological arousal and consciously perceived feelings of depression, tension, and apprehension (Spielberger et al., 1983; Endler and Kocovski, 2001). There were also some studies considering state anxiety as the state associated with the feeling of anxiety and considering trait anxiety as the frequency of anxiousness (Takagi et al., 2018). State anxiety and trait anxiety were correlated, but independent behavioral measurements that had different influences on attentional and cognitive control processes (Bishop et al., 2007; Crocker et al., 2012; Hur et al., 2015), and distinguishment between them was important for evaluating and monitoring individuals' levels of anxiety as well as developing effective means to prevent and treat anxiety-related disorders (Forrest et al., 2021). Although state and trait anxiety have independent behavioral definitions, it remained unclear whether they shared common or distinct brain network topological mechanisms.

In the neuroscience field, a large number of studies have investigated the neural underpinnings of state and trait anxiety, either common or distinct, using different experimental approaches and techniques. While most studies concluded that they had distinct neuroanatomical and functional substrates (Satpute et al., 2012; Tian et al., 2016; Saviola et al., 2020). In more detail, at the structural level, trait anxiety was associated with gray matter volume in the frontal cortex (Hu and Dolcos, 2017) and the occipital gyrus (Yin et al., 2016), as well as altered cortical thickness in the temporal cortex, the cingulate cortex, and the orbitofrontal cortex (Potvin et al., 2015); at the functional level, trait anxiety was related to regional homogeneity (ReHo) or amplitude of low-frequency fluctuations (ALFF) in the prefrontal cortex (PFC), cingulate cortex (Tian et al., 2016), orbitofrontal cortex (Xue et al., 2018), thalamus, and cerebellum (Yin et al., 2016), as well as functional connectivity (FC) in brain networks, including default mode network (DMN) (Modi et al., 2015; Saviola et al., 2020), ventral attention network (He et al., 2016), and temporo-parietal-frontal network (Modi et al., 2015; He et al., 2016). As for state anxiety, neuroimaging studies concerning structural gray matter volume or thickness are lacking (Saviola et al., 2020) and functional substrates in state anxiety mainly involved limbic regions such as the insula (Tian et al., 2016) and hippocampus (Satpute et al., 2012) in different anxiety provocation tasks. However, there were also several studies showing that there was an interaction and association between state and trait anxiety (Mathews, 1990; Williams et al., 1996). From a systematical perspective, Takagi et al. (2018) found a common brain network among state and trait anxiety in a unified analytical framework. Meanwhile, different experimental approaches revealed that state and trait anxiety were correlated with the same brain regions such as the PFC (Mataix-Cols et al., 2003; Tian et al., 2016), occipital gyrus (Yin et al., 2016; Li et al., 2020), as well as parietal-frontal network (Modi et al., 2015; He et al., 2016; Li et al., 2020). In summary, although previous studies demonstrated that state and trait anxiety shared common or distinct neural substrates, few studies provided a convincing conclusion from multiple spatial and temporal scales, as well as in both brain structure and brain function (Saviola et al., 2020).

Complex network analysis could be used to explore human brain network topological properties and enhance our comprehension on human brain network architecture and their associations with behaviors (Bullmore and Sporns, 2009; Sporns, 2011). Brain functional network construction was mainly based on FC (temporal correlation of brain activity) between brain regions (nodes) (Bullmore and Sporns, 2009) using electrophysiological or functional imaging techniques. Previous studies mostly calculated static FC that utilized all the time points (Salvador et al., 2005; He et al., 2016; Saviola et al., 2020). As temporal variability in fMRI signals has already been detected across a typical course of a single scan (Chang and Glover, 2010; Kang et al., 2011; Hutchison et al., 2013a), BOLD signal correlations would definitely show dynamic changes over time scales of seconds to minutes (Chang and Glover, 2010). Also, this dynamical nature of FC reflected a basic property of complex systems such as human brain (Liu and Duyn, 2013; Liao et al., 2015). Therefore, in this study, we would combine static and dynamic FC to construct brain functional network. In contrast, existing methods to construct brain structural network are mainly white matter tractography (Li et al., 2009; Koenis et al., 2018) and structural covariance network (Mechelli et al., 2005; Alexander-Bloch et al., 2013). As stated in our previous study (Li et al., 2021), the white matter tractography could not reliably quantify long-range structural connectivity (Jeurissen et al., 2019) and was largely affected by head motion and might involve a large number of false-positive connections (Thomas et al., 2014; Maier-Hein et al., 2017); the structural covariance network from a large number of participants only yielded one single correlation matrix but did not reveal individual differences (Alexander-Bloch et al., 2013; Li et al., 2021). Therefore, in this study we still used macroscale morphology network that was based on distributions of cortical surface characteristics (cortical volume, thickness, and surface area) from resting-state fMRI (Li et al., 2021) in order to explore the neural substrates of state and trait anxiety. Corresponding to the temporal variations of human brain functional network, we changed bin number of frequency distributions of cortical surface characteristics (area, thickness, and volume) to explore the spatial variations of brain structural network. We expected that topological properties of brain structural and functional networks, as well as their spatial and temporal variations, would efficiently distinguish state anxiety from trait anxiety.

Network topology means the full connection details of a network, that is, human brain connectome (Bullmore and Sporns, 2009; Rubinov and Sporns, 2010). Centrality and efficiency are two commonly used topological measures in human brain network studies. Centrality assesses the importance of a brain region (node) in facilitating functional integration and interaction across the entire network architecture (Rubinov and Sporns, 2010; Zuo et al., 2012). As for efficiency, Latora and Marchiori (2001) demonstrated that network efficiency could be used to measure the information flow within human brain network: global efficiency corresponds to long-distance interactions and reflects information integration over the whole network; nodal efficiency reflects information transfer ability of the parcel (node); whereas local efficiency reflects specialization of a single brain region (node) within the network (Latora and Marchiori, 2001; Rubinov and Sporns, 2010; Bullmore and Sporns, 2012). Global efficiency, nodal efficiency, as well as betweenness centrality, eigenvector centrality, and pagerank centrality characterized functional integration across the network architecture, while local efficiency and degree centrality focus on the information flow and transfer in local brain regions and functional segregation (Rubinov and Sporns, 2010). These topological properties have been used to successfully detect the neural basis of various behaviors and diseases (Zuo et al., 2012; Guo et al., 2016; Weiler et al., 2018; Li et al., 2021), did state and trait anxiety share a common or distinct brain network topological mechanism in both functional integration and segregation perspectives?

To determine whether state anxiety and trait anxiety shared a common or distinct topological mechanism of human brain structural and functional networks, we recruited 67 healthy participants who finished structural and resting-state fMRI scanning, followed by the assessments of state and trait anxiety. We respectively, used morphological similarity and FC method to construct human brain structural and functional networks and aimed to elucidate the topological mechanisms of state and trait anxiety from both the single-network perspective and their spatial and temporal variations perspective (Allen et al., 2014). Our study confirmed that state and trait anxiety shared common human brain network topological mechanisms in the insula and the frontal eyes field, which were involved in preliminary cognitive processing stage of anxiety. Our study also demonstrated that the common brain network topological mechanisms had high spatiotemporal robustness and would enhance our understanding of human brain temporal and spatial organization.

## Methods

### Participants

Participants were recruited from local community or universities by advertisements, and the initial sample included 67 datasets (32 males and 35 females; mean age = 32.79 ± 13.11; ranged from 18.59 to 64.30). All the participants underwent a detailed mental health interview by two trained psychologists using the Mini-International Neuro-Psychiatric Interview. People with a history of major neuropsychiatric illness, head injury, and alcohol and drug abuse were excluded. They were also assessed with Wechsler Adult Intelligence Scale-4th Edition (in Chinese, WAIS-IV), Schutte Self-Report Emotional Intelligence scale in Chinese Version (SSEIS), State-Trait Anxiety Inventory, Mental Health Continuum-Short Form, Emotion Regulation Questionnaire, Chinese Perceived Stress Scale, Achievement Motivation Scale, and Self-Control Scale. The institutional review board of Institute of Psychology Chinese Academy of Sciences approved this study, and written informed consent was obtained from individual participant prior to data acquisition.

### Behavior Measures

The State-Trait Anxiety Inventory (STAI) (Spielberger et al., 1983) was applied to measure participants' state and trait anxiety. The scale has a total of 40 descriptive questions related to anxiety. Items 1–20 are the State Anxiety Scale (S-Al), 10 of which describe negative emotions and 10 of which describe positive emotions, which are used to assess the feelings of people at a specific and particular moment. Items 21–40 are the Trait Anxiety Inventory (T-AI), 11 of which describe negative emotions and 9 of which describe positive emotions, which are used to assess people's habitual anxiety experience and capture the dimensions of personality linked to anxiety. Each item uses a 4-point scoring method. The higher scores indicate the higher degree of anxiety. Previous studies have verified that the Chinese version of the STAI had acceptable construct validity that supported Spielberger's conception of the multidimensional nature of the S-AI and T-AI scales (Li and Lopez, 2004; Wei et al., 2015) and could be used to measure state and trait anxiety of Chinese participants. Cronbach's α coefficient for internal consistency in our sample was acceptable (state anxiety α = 0.757, trait anxiety α = 0.717).

### Imaging Acquisition

MRI images were collected on the 3.0 T GE scanner Discovery MR750 at the Institute of Psychology Chinese Academy of Sciences. All the participants completed a T1-weighted structural MRI scan (eyes closed) with a ABI1_t1iso_fspgr sequence (TR = 6.652 ms; TE = 2.928 ms; FA = 12°; matrix = 256 × 256; slice thickness = 1 mm) and an 8-min resting-state fMRI scan (eyes open with a fixation cross) using a gradient echo EPI sequence ABI1_bold_bw_rest [TR = 2,000 ms; TE = 30 ms; FA = 90°; number of slices = 33 (interleaved); slice thickness = 3.5 mm; gap = 0.7 mm; and matrix = 64 × 64].

### Imaging Data Preprocessing

MRI images were preprocessed using the Connectome Computation System (http://github.com/zuoxinian/CCS) developed by our laboratory (Xu et al., 2015), which integrated several neuroimaging-related software including AFNI (Cox, 2012), FSL (Jenkinson et al., 2012), and FreeSurfer (Fischl, 2012), as well as in-house MATLAB scripts. The full pipeline of preprocessing included structural image preprocessing, functional image preprocessing, as well as quality control (Zuo et al., 2013; Xu et al., 2015). The structural preprocessing included (1) intensity inhomogeneity correction; (2) brain extraction; (3) tissue segmentation; (4) white and pial surface generation; and (5) deformation estimation between the resulting spherical mesh and a common spherical coordinate system. The functional preprocessing was the same as our previous publications (Jiang et al., 2015; Zhang et al., 2017) and included (1) excluding the first five volumes from each scan; (2) removing and interpolating of temporal spikes; (3) slice timing correction and motion correction; (4) extracting functional brain; (5) normalizing 4D global mean of image intensity; and (6) co-registration between functional and anatomical images by employing a boundary-based registration (BBR) algorithm.

### Quality Control Procedure

Following the preprocessed individual MRI images, the CCS also provided a quality control procedure (QCP) for both functional and structural images. The QCP includes the following steps (Zuo et al., 2013): (1) brain extraction or skull stripping; (2) brain tissue segmentation; (3) pial and white surface reconstruction; (4) BBR-based functional image registration; and (5) head motion during resting-state MRI. The pipeline also computed the mean frame-wise displacement (meanFD) (Power et al., 2012) and the minimal cost of the BBR co-registration (mcBBR) for the subsequent statistical tests as covariates. All participants with bad brain extraction, bad tissue segmentation, and bad surface construction will be excluded from the subsequent analysis. One participant did not complete MRI scanning, and one participant did not pass the mental health interview. Five participants were excluded because their mcBBR was >0.65. Therefore, we had 60 participants for final group analysis. The detailed participant information and intervariable correlations are shown in Table 1.

**Table 1:** Participant information: descriptive statistics and inter-variable correlations.

**Variable**	**Mean**	**SD**	**Age**	**Education**	**State anxiety**
Age	37.81	13.10	1		
Education	15.48	3.10	−0.464^**^	1	
State anxiety	34.20	9.33	−0.160	0.087	1
Trait anxiety	34.63	9.24	−0.177	0.106	0.848^**^

*N = 60; SD, standard deviation*;

** *p < 0.01*.

### Brain Network Construction

Here, we used a macroscale brain network parcellation developed by Yeo et al. (2011) as nodes to construct human brain structural and functional network, which subdivided the entire cortical surface into 51 spatially connected parcels based on resting-state FC. We excluded the parcels whose vertex number was <50, and finally 30 parcels were reserved for final group analysis, which expanded across all the Yeo-7 networks, including visual network, somatomotor network, dorsal attention network, ventral attention network, limbic network, frontoparietal (control) network, and DMN (Table 2).

**Table 2:** Information of brain regions reserved in our network analysis (vertex number > 50).

**Brain region (LH)**	**Vertex number**	**Brain region (RH)**	**Vertex number**
Vis	1,213	Vis	1,262
SomMot	1,590	SomMot	1,612
DorsAttn_Post	616	DorsAttn_Post	589
DorsAttn_FEF	97	DorsAttn_FEF	98
SalVentAttn_ParOper	130	DorsAttn_PrCv	50
SalVentAttn_FrOper	331	SalVentAttn_TempOccPar	208
SalVentAttn_Med	216	SalVentAttn_FrOper	313
Limbic_TempPole	164	SalVentAttn_Med	242
Cont_Par	151	Limbic_TempPole	136
Cont_PFCl	291	Cont_Par	167
Default_Par	263	Cont_PFCl	537
Default_Temp	311	Default_Par	183
Default_PFC	768	Default_Temp	246
Default_PCC	275	Default_PFCv	60
		Default_PFCm	453
		Default_PCC	225

#### Morphological (Structural) Network

**Figure 1:**
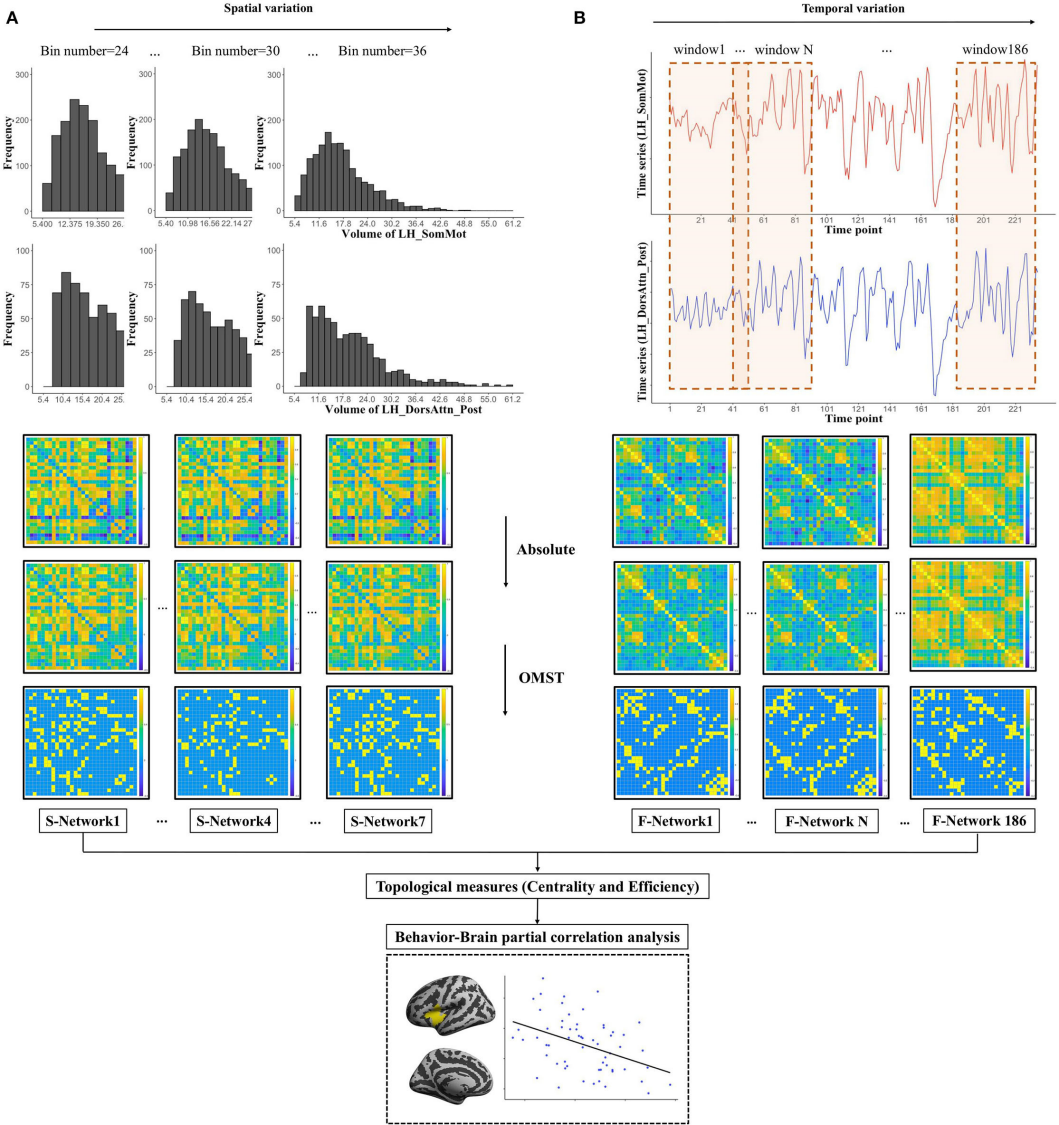
The workflow for the construction and thresholding of morphological (structural) and functional network. **(A)** For each pair of parcels such as the left SomMot and the left DorsAttn_Post of right hemisphere, we uniformly divided their volumes into 30 bins (5.4–61.2). Each bin will have the width of (61.2–5.4)/30 = 1.68, and the frequency distribution histograms are presented in the upper. By computing the Pearson's correlation of two sets of frequencies, we got the similarity of these two parcels. Then, we changed the bin number from 24 to 36 with a step of 2 bins to get 7 structural networks. **(B)** For each pair of parcels such as the left SomMot and the left DorsAttn_Post of right hemisphere, we used the sliding window method and segmented the total 235 time points of each parcel into windows of 50 TRs (100 s) shifting with a step size of 1 TR to obtain 186 functional networks. And then we showed the thresholding schemes of the network: we first got absolute value of each connection in the network and then applied the data-driven thresholding scheme based on orthogonal minimal spanning trees (OMST) to get the strongest and the most important connections. Finally, we calculated the topological measures based on the connection networks to explore the correlations between anxiety and human brain topological properties.

We have proposed a new method to construct structural network, including cortical volume, surface area, and cortical thickness, in our previous study (Li et al., 2021), which assessed the distribution similarity of each morphological measurement for each pair of parcels (Figure 1A), consider the cortical volume as an example). First, for each pair of parcels, we uniformly divided their volumes into 30 bins. Second, we computed the vertex frequency for each bin of the parcels, so that we got the frequency distribution histogram for each parcel. Third, the Pearson correlation coefficient of the frequency distribution histograms was calculated to estimate the volume distribution similarity, and we got a 30 → 30 morphological correlation matrix for each participant. Considering the effects of bin number on network architecture, we traversed the bin number from 24 to 36 shifting with a step size of 2 bins to get 7 structural networks to assess the spatial variations of human brain structural network.

#### Functional Network

For each parcel of cortical surface, we calculated an average time sequence (235 time points), and then we computed the Pearson correlation coefficient of time series on each pair of parcels to get a 30 → 30 correlation matrix for each participant. The sliding window approach (Allen et al., 2014) was then used to calculate the dynamic FC between each pair of parcels to assess the temporal variations of human brain functional network (see Figure 1B). A total of 50 TRs were selected as the window length, on the one hand, which covered the low-frequency band of interest (0.01–0.1 Hz) with an adequate number of time points (at least one period) (Liao et al., 2015), on the other hand, which optimized the balance between the specificity (long enough to detect convincing dynamic fluctuations) and sensitivity (short enough to permit real dynamic variations) of dynamic functional connections (He et al., 2018; Li et al., 2018; Guo et al., 2020). Finally, the total 235 time points of each parcel were segmented into windows of 50 TRs (100 s) shifting with a step size of 1 TR, and we obtained 186 functional networks. We also have tried other window lengths (60 and 70 TRs), and our conclusions were not changed.

After constructing brain structural and functional networks, we used orthogonal minimal spanning trees [OMST, (Dimitriadis et al., 2017)], which was a threshold-free method to extract the strongest and the most important connections of a network, to get an undirected weighted graph. Then, we computed topological properties of human brain network, network efficiency and centrality, and the whole pipeline of data analysis is shown in Figure 1.

### Topological Measures

Network topology means the full details of network connections, and here we mainly considered two types of topological measurements, namely, network efficiency and centrality. In more detail, we applied graph theory (Achard et al., 2006) to compute network efficiency, including global efficiency (*Eglob*), nodal efficiency (*Enodal*), and local efficiency (*Elocal*), as well as network centrality, including degree centrality (*DC*), betweenness centrality (*BC*), eigenvector centrality (*EC*), and pagerank centrality (*PC*), and the specific calculation tools included the Brain Connectivity Toolbox (http://www.brain-connectivity-toolbox.net) (Rubinov and Sporns 2010) and the Connectome Computation System (http://github.com/zuoxinian/CCS) scripts (Xu et al., 2015).

#### Network Efficiency

Global efficiency for a network G is defined as follows:


(1) $$\begin{array}{r} E_{glob}\left(G\right)=\frac{1}{N\left(N-1\right)}\underset{i,j,i\neq j\in G}{\sum }\frac{1}{L_{ij}} \end{array}$$

where *N* is the number of nodes (brain regions) and *L*_*ij*_is the shortest path length between node *i* and node *j* in network G (Latora and Marchiori, 2003). Global efficiency refers to the overall information transfer efficiency between any two nodes (brain regions) in the whole brain, which is a long-distance information transmission in the network and reflects a global measure of the information transmission efficiency of the whole network (Latora and Marchiori, 2003).

Nodal efficiency of node *i* is defined as follows:


(2) $$\begin{array}{r} E_{nodal}\left(i\right)=\frac{1}{N-1}\underset{j,i\neq j\in G}{\sum }\frac{1}{L_{ij}} \end{array}$$

where *N* and *L*_*ij*_ are the same as that in Equation (1). The nodal efficiency represents the importance of the node for information transfer in the network, and the global efficiency is the average of the node efficiency of all nodes (brain regions) in the whole brain.

Local efficiency of node *i* is defined as follows:


(3) $$\begin{array}{r} E_{local}\left(i\right)=E_{glob}\left(G_{i}\right) \end{array}$$

where *G*_*i*_ is a subgraph and composed of the nodes that connect to node *i* (not including node *i*) directly and interconnected edges. The average of the reciprocals of the shortest paths between any two nodes within the subgraph is the local efficiency. Local efficiency indicates the information transfer ability in the given subgraph, and more densely clustered connections between topological neighbors indicated higher local efficiency (Latora and Marchiori, 2003; Bullmore and Sporns, 2012).

#### Network Centrality

Degree centrality of node *i* is defined as follows:


(4) $$\begin{array}{r} DC\left(i\right)=\underset{j\in G}{\sum }a_{ij} \end{array}$$

where *a*_*ij*_ is the connection status between *i* and *j*: *a*_*ij*_ = 1 when i and j are connected and *a*_*ij*_= 0 when i and j are not connected. DC is the number of links connected to a node and represents the most local and directly quantifiable centrality measure, and higher DC of the node indicates more important role in the network (Rubinov and Sporns, 2010; Zuo et al., 2012).

Betweenness centrality of node *i* is defined as follows:


(5) $$\begin{array}{r} BC\left(i\right)=\underset{h,j\in G,\;h\neq j,h\neq i,j\neq j}{\sum }\frac{L_{hj}\left(i\right)}{L_{hj}} \end{array}$$

where *L*_*hj*_ is the number of shortest paths between node *h* and node *j*, and *L*_*hj*_*(i)* is the number of shortest paths between *h* and *j* that pass through node *i*. BC represents the fraction of all shortest paths in the network that pass through a given node. Important and bridging nodes that connect disparate parts of the network exhibited a high BC (Freeman, 1979; Rubinov and Sporns, 2010).

Eigenvector centrality of node *i* is defined as follows:


(6) $$\begin{array}{r} EC\left(i\right)=\mu_{1}\left(i\right)=\frac{1}{\lambda_{1}}\overset{N}{\underset{j=1}{\sum }}a_{ij}\mu_{1}\left(j\right) \end{array}$$

where μ_*j*_(*i*) is the *i*th component of the *j*th eigenvector of the adjacency matrix *a*_*ij*_, and λ_1_ is the corresponding *j*th eigenvalue. N is the number of nodes, and *a*_*ij*_ is the association matrix. Nodes have high EC if they connect to other nodes that have high EC, which means higher EC scores indicate a more central and important role of the node in the network (Bonacich, 1972; van Duinkerken et al., 2017).

Pagerank centrality of node *i* is defined as follows:


(7) $$\begin{array}{r} PC\left(i\right)=r\left(i\right)=1-d+d\overset{N}{\underset{j=1}{\sum }}\frac{a_{ij}r\left(j\right)}{DC\left(j\right)} \end{array}$$

The pagerank measure was introduced originally by Google to rank web pages. In the graph theory, Pagerank represents the “importance” of nodes assuming that the importance of a node is the expected sum of the importance of all connected nodes and the direction of edges (Gleich, 2015; Henni et al., 2018). The Google pagerank centrality algorithm is a variant of EC, which introduces a small probability (1–d = 0.15, d is damping factor) of random damping to handle walking traps on a graph (Boldi et al., 2009).

Besides the above topological properties themselves, we also calculated the coefficient of variation (CV) of the topological properties to explore the temporal and spatial variations underlying state and trait anxiety. Meanwhile, we divided the value of topological measures in 186 functional brain networks into frequency distribution histograms whose bin number was 20 and calculated medians, modes, and half width at half maxima (FWHMs) of the histograms, as well as medians, modes, and FWHMs of the histogram-fitted curves.

### Statistics

To investigate topological mechanisms of human brain structural and functional networks underlying state and trait anxiety, we used general linear model to calculate the partial correlations between topological properties (E, efficiency; C, centrality; CV, coefficient of variation) and anxiety. In more detail, for structural network, we considered age, gender, education, intracranial volume (ICV), and total volume for volume network (total area for area network or mean thickness for thickness network) as covariates. The detailed statistical model is shown in the following equation:


(8) $$\begin{array}{l} Anxiety=\alpha_{1}\times age+\alpha_{2}\times gender+\alpha_{3}\times education\\ \;+\alpha_{4}\times ICV+\alpha_{5}\times morp_{mean/total}+\beta \times E/C/CV\\ \;+\gamma \end{array}$$

For functional network, we considered age, gender, education, mcBBR, and meanFD as covariates. The detailed statistical model is shown in the following equation:


(9) $$\begin{array}{l} Anxiety=\alpha_{1}\times age+\alpha_{2}\times gender+\alpha_{3}\times education\\ \;+\alpha_{4}\times mcBBR+\alpha_{5}\times meanFD+\beta \times E/C/CV\\ \;+\gamma \end{array}$$

False discovery rate (FDR, *q* < 0.05) correction for 30 parcels was used to control type 1 error over multiple tests, and all the statistical analyses were performed using MATLAB scripts in this study.

## Results

Table 1 presents detailed information about state-anxiety and trait-anxiety for the entire group of participants, including their average and standard deviation. There was no significant correlation between anxiety and demographic variables such as age and education. However, state and trait anxiety were significantly correlated, which demonstrated that the two dimensions of anxiety might share some common components.

### State and Trait Anxiety Were Both Associated With Topological Characteristics of Human Brain Structural and Functional Networks

**Figure 2:**
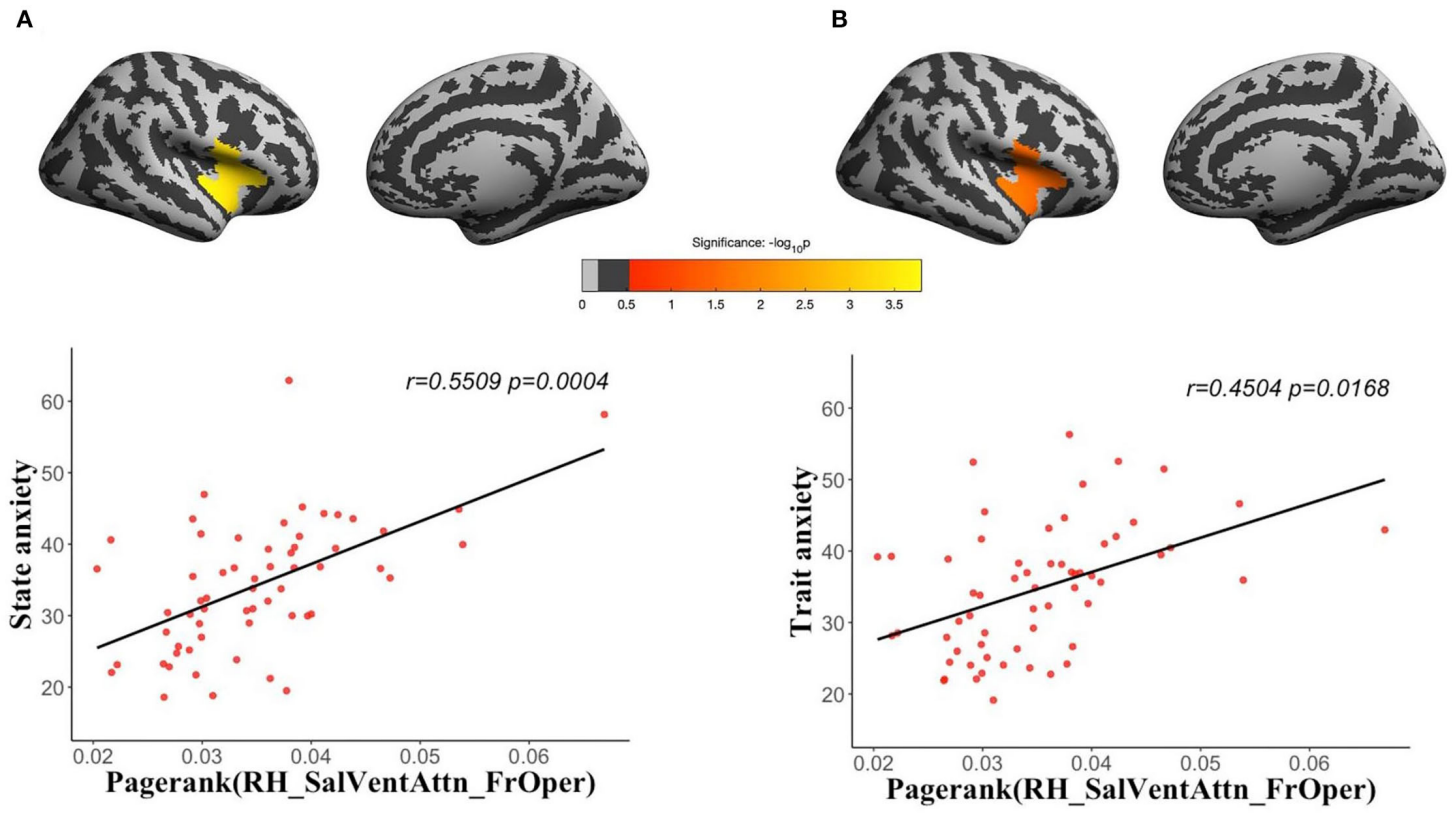
Brain regions with significant correlations between topological properties of human brain network and anxiety. Pagerank centrality in the RH_SalVentAttn_FrOper (the opercular part of the inferior frontal gyrus, the inferior and superior insula of right hemisphere) of human brain functional networks was not only positively correlated with state anxiety [**(A)** *r* = 0.5509, corrected *p* = 0.0004], but also was positively correlated with trait anxiety [**(B)** *r* = 0.4505, corrected *p* = 0.0168].

We used morphological similarity network (bin number = 30) of cortical volume, surface area, cortical thickness, and FC network (time point = 235) to explore whether state anxiety and trait anxiety had common or distinct topological mechanisms. For functional network, we found state anxiety (*r* = 0.5509, *corrected p* = 0.0004) and trait anxiety (*r* = 0.4505*, corrected p* = 0.0168) were both significantly positively correlated with pagerank centrality in the RH_SalVentAttn_FrOper (Figures 2A,B), which mapped to the opercular part of the inferior frontal gyrus, and the inferior and superior of the insula (Destrieux et al., 2010).

### State and Trait Anxiety Shared Common Topological Mechanisms Based on Spatial Variations of Human Brain Structural Network and Temporal Variations of Human Brain Functional Network

Using different spatial scales during the construction of human brain morphological similarity network, we got spatial variations of human brain structural network. Using sliding-window method, we got temporal variations of human brain functional network. The detailed topological properties for different spatial scales and different time slots are illustrated in Supplementary Figure 1.

**Figure 3:**
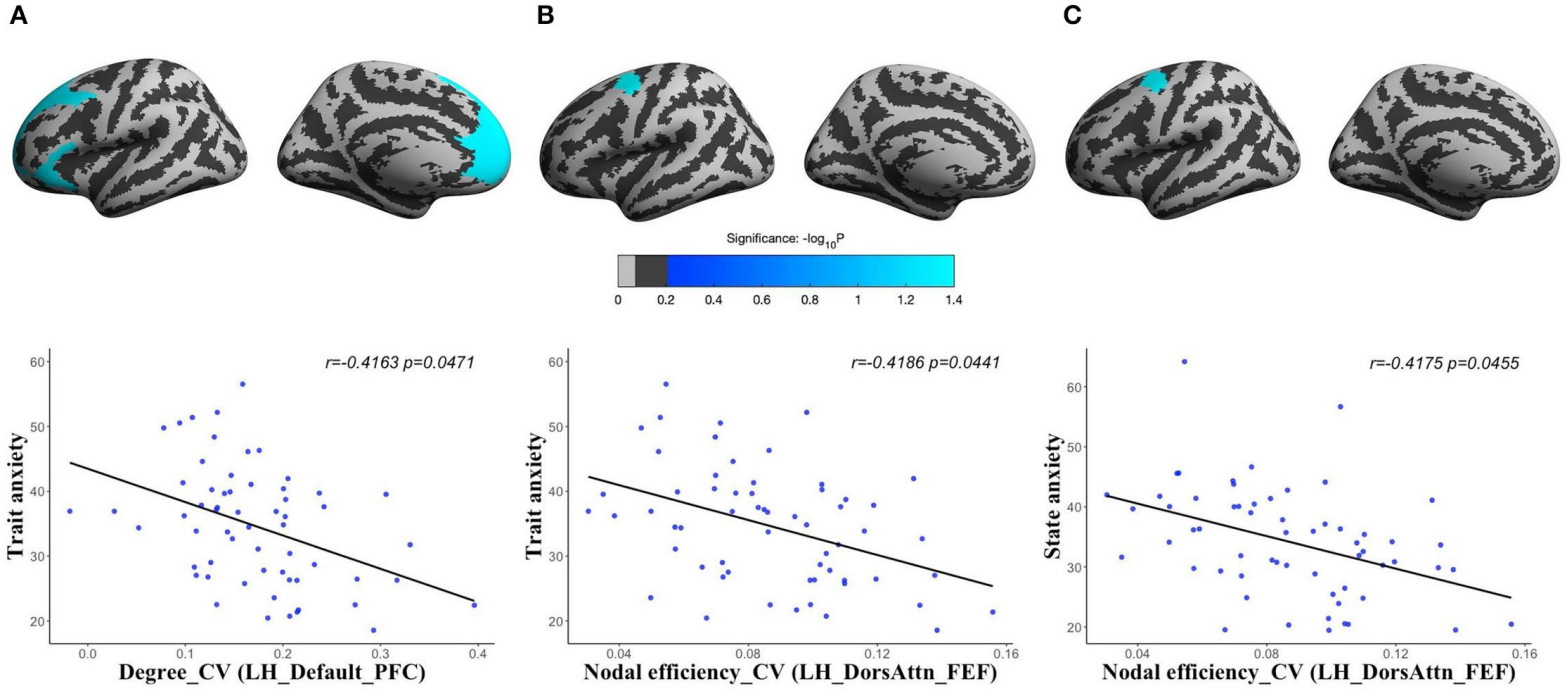
The partial correlations between the spatial variations of human brain structural networks and anxiety: CV of degree centrality in the LH_Default_PFC (the PFC of left hemisphere) of volume network with trait anxiety [**(A)** *r* = −0.4163, corrected *p* = 0.0471]; CV of nodal efficiency in the LH_DorsAttn_FEF (the frontal eyes field of left hemisphere) of volume network with trait anxiety [**(B)** *r* = −0.4186, corrected *p* = 0.0441]; and CV of nodal efficiency in the LH_DorsAttn_FEF of volume network with state anxiety [**(C)** *r* = −0.4175, corrected *p* = 0.0455].

For human brain structural network at different spatial scales, we found that the CV of degree centrality was negatively correlated with trait anxiety in the LH_Default_PFC (corresponding to PFC) (*r* = −0.4163, *corrected p* = 0.0471), and CV of nodal efficiency was significantly negatively correlated with both state anxiety (*r* = −0.4175, *corrected p* = 0.0455) and trait anxiety (*r* = −0.4186, *corrected p* = 0.0441) in the LH_DorsAttn_FEF (corresponding to frontal eyes field) of volume network (Figure 3). There were no significant results in other morphological networks.

**Figure 4:**
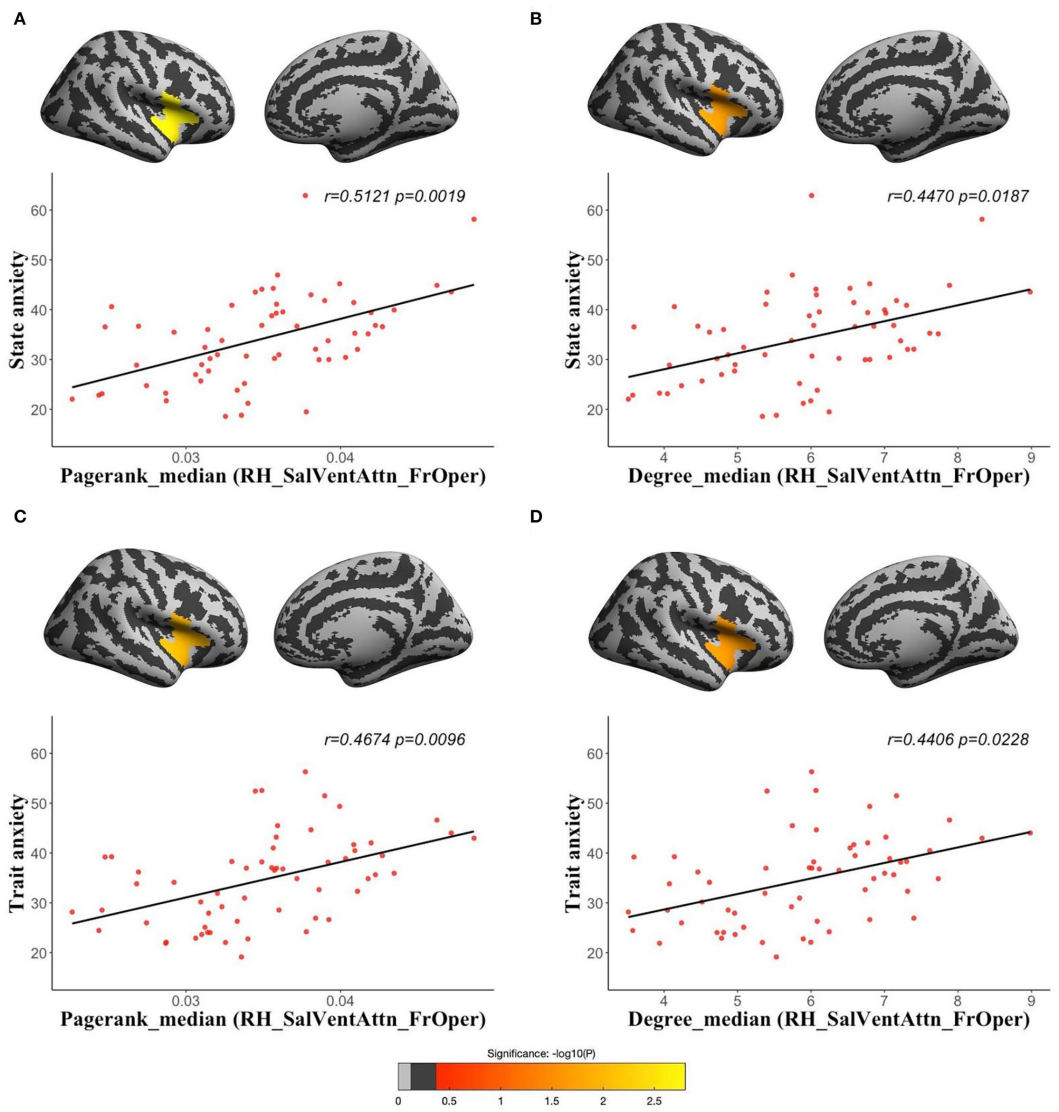
The partial correlations between the temporal variations of topological measures of human brain functional networks and anxiety: median of pagerank centrality in the RH_SalVentAttn_FrOper (the opercular part of the inferior frontal gyrus, the inferior and superior insula of right hemisphere) with state anxiety [**(A)** *r* = 0.5121, corrected *p* = 0.0019]; median of degree centrality in the RH_SalVentAttn_FrOper with state anxiety [**(B)** *r* = 0.447, corrected *p* = 0.0187]; median of pagerank centrality in the RH_SalVentAttn_FrOper with trait anxiety [**(C)** *r* = 0.4674, corrected *p* = 0.0096]; and median of degree centrality in the RH_SalVentAttn_FrOper with trait anxiety [**(D)** *r* = 0.4406, corrected *p* = 0.0228].

Using dynamic functional networks acquired from sliding window, we found both state and trait anxiety were positively correlated with median of pagerank centrality (state anxiety: *r* = 0.5121, *corrected p* = 0.0019; trait anxiety: *r* = 0.4674, *corrected p* = 0.0096) and median of degree centrality (state anxiety: *r* = 0.447, *corrected p* = 0.0187; trait anxiety: *r* = 0.4406, *corrected p* = 0.0228) in the RH_SalVentAttn_FrOper (Figure 4). We also found both state and trait anxiety were positively correlated with median of betweenness centrality (state anxiety: *r* = 0.3991, *corrected p* = 0.0381; trait anxiety: *r* = 0.4322, *corrected p* = 0.0295) and mode of degree centrality (state anxiety: *r* = 0.4289, *corrected p* = 0.0326; trait anxiety: *r* = 0.4308, *corrected p* = 0.0308) in the RH_SalVentAttn_FrOper (Supplementary Figure 3); trait anxiety was positively associated with median (*r* = 0.3991, *corrected p* = 0.0381) and mode (*r* = 0.4265, *corrected p* = 0.0325) of pagerank centrality in the LH_SalVentAttn_FrOper, median (*r* = 0.4104, *corrected p* = 0.0381) and FWHM (*r* = 0.4242, *corrected p* = 0.0360) of betweenness centrality in the LH_SalVentAttn_FrOper, and mode (*r* = 0.4909, *corrected p* = 0.0325) of pagerank centrality in the RH_SalVentAttn_FrOper (Supplementary Figure 2). Besides the above parcel-wise network, we also examined vertex-wise functional network and got similar results as shown in Supplementary Table 1. We also tried group comparisons between high-anxiety and low-anxiety groups, and found that both state and trait anxiety were associated with global efficiency of area similarity network (Supplementary Tables 2–8).

## Discussion

To examine whether state and trait anxiety share common or distinct neural substrates, we conducted systematical studies on the associations of anxiety with topological properties of human brain network, including morphological similarity network and its spatial variations, as well as FC network and its temporal variations, from both parcel-wise and vertex-wise perspectives. Our results showed that (1) in the static functional network, state and trait anxiety were both positively correlated with pagerank centrality in the right insula; (2) in the dynamic functional network, state and trait anxiety were both positively correlated with median and mode of pagerank and degree centrality in the right insula; and (3) state and trait anxiety were both negatively correlated with CV of nodal efficiency in the left frontal eyes field of volume network. In summary, our results demonstrated that there might be a common neural and topological mechanism between state and trait anxiety in different network conditions.

### The Roles of the Insula and the FEF in Anxiety

At the human brain functional level, we found that pagerank centrality of the anterior insula, which mostly occupied the right frontal operculum (RH_SalVentAttn_FrOper), was significantly positively correlated with state and trait anxiety in both static and dynamic functional networks. This was in line with previous studies indicating the activations of the insula in healthy participants' anxiety provocation tasks (Tian et al., 2016; Geng et al., 2018; Dammann et al., 2020). The insula has been proved to play an important role in subjective emotional processing and internal body awareness (Critchley et al., 2004; Craig, 2009; Singer et al., 2009; Tranel et al., 2009; Ernst et al., 2013), anticipation of future uncertain threat (Geng et al., 2018) and unpleasant negative stimuli (Herwig et al., 2007; Simmons et al., 2011; Lutz et al., 2013), risk and uncertainty in decision-making (Grinband et al., 2006; Clark et al., 2008), as well as visual and auditory awareness (Bushara et al., 2001; Kondo and Kashino, 2007). The positive correlations between topological properties of the insula and anxiety indicated that healthy participants with higher state and trait anxiety might be prone to pay more attention and awareness to internal body feelings and anticipate the risky and uncertain future events or threats due to the activations of the insula. Meanwhile, the role of the insula was also reflected in anxiety disorders, including social anxiety disorder (Duval et al., 2018; Atmaca et al., 2021) and generalized anxiety disorder (Shah et al., 2009; Cui et al., 2020), where they found more interoceptive body awareness, deficits in attentional control and emotion modulation, as well as hyperactive to general emotional images in patients compared to healthy controls. All these studies together demonstrated the connections between the insula and anxiety.

While at the human brain structural level, we found that spatial variations (CV) of nodal efficiency in the left frontal eyes field (FEF) was negatively correlated with state and trait anxiety, which reflected that decreased information transfer ability and efficiency of the FEF contributed to higher state and trait anxiety. The FEF, located in the superior precentral sulcus of the superior frontal sulcus corresponding to Brodmann's area 6 (Vernet et al., 2014), was not only involved in preparing and triggering various eye movements (Nyffeler et al., 2004; Nagel et al., 2008; Yang and Kapoula, 2011; Jaun-Frutiger et al., 2013), but also important in cognitive processes, including attentional orienting (Muggleton et al., 2003; Grosbras et al., 2005), visual awareness (Smith et al., 2005; Quentin et al., 2013), and perceptual modulation (Thompson et al., 1996). The negative correlations indicated participants with high state and trait anxiety had weakened ability and efficiency of attention control as well as perceptual modulation, and might show a tendency to increase more attention to unpleasant threats and stimuli, which was consistent with the attention control theory (Eysenck et al., 2007).

### State and Trait Anxiety Shared Common Topological Mechanisms of Human Brain Networks

In this study, we conducted systematical studies to confirm that state and trait anxiety shared common brain network topological mechanisms. We found topological properties of the insula and the FEF were both correlated with state and trait anxiety, respectively, at human brain functional and structural level, which suggested that healthy participants who tended to feel anxious in daily life (trait anxiety) might share the same brain network topological patterns during anxious events and threats evocation (state anxiety). Previous studies have demonstrated that there was an interaction and association between state anxiety and trait anxiety (Mathews, 1990; Williams et al., 1996). More importantly, Takagi et al. (2018) have directly demonstrated that trait and state anxiety shared the same brain network and had a substantial biological interrelationship in a unified analytical framework. They hypothesized that participants with high trait anxiety would become increasingly anxious when facing unanticipated anxiety-related events and further enhance their levels of state anxiety. In contrast, participants with low trait anxiety would show a resistant and defensive response to the threats to reduce their levels of state anxiety. In more detail, the insula and the FEF were both involved in preliminary cognitive processing stage awareness (whether external or internal; visual or auditory), which indicated that state and trait anxiety shared the same brain topology mechanisms in alarming and sensing anxiety-related signals. Meanwhile, we also found that spatial variations (CV) of degree centrality in the PFC were only negatively correlated with trait anxiety but not state anxiety. The PFC generally acts as a role in emotion regulation and generation (Dixon et al., 2017), as well as integrates internal and external stimuli, to influence behavioral reactions (Bickart et al., 2012; Lindquist et al., 2012). We inferred that state and trait anxiety might use different human brain network topological mechanisms in the subsequent processes of coping with anxiety-related threats or events.

### Temporospatial Robustness of the Common Topological Mechanisms of Human Brain Networks Between State and Trait Anxiety

To the best of our knowledge, we are the first to not only explore the topological properties underlying state and trait anxiety using MRI technique but also verify that the common topological properties of human brain structural and functional networks underlying state and trait anxiety had temporospatial robustness. Human brain is one of the most complicated systems in the world, and it must coordinate complicated information at different spatial and temporal scales. Based on temporal correlations of BOLD time series, functional network could be used to detect human brain functional organization at different temporal scales (Friston, 2011; Allen et al., 2014; Calhoun Vince et al., 2014). Traditional FC method employed correlation coefficient of the time series of the entire scan across brain regions without capturing the temporal variations, while dynamic FC could quantify temporal alterations in FC metrics across multiple temporal scales (Hutchison et al., 2013b). Dynamic FC was proved to be a sensitive and specific marker of mental illness (Calhoun Vince et al., 2014; Yao et al., 2017). In more detail, previous studies have applied dynamic FC to various diseases such as generalized anxiety disorder (Yao et al., 2017; Chen et al., 2020), schizophrenia (Damaraju et al., 2014; Du et al., 2017), and bipolar disorder (Rashid et al., 2016; Nguyen et al., 2017). All these validated that dynamic FC was useful and efficient in characterizing human brain functional organization at different temporal scales. Corresponding to functional network, we also changed bin number (spatial scales to measure morphological distribution) and gained different morphological similarity resolutions to investigate spatial variations of human brain structural network underlying state and trait anxiety. Previous studies have demonstrated that morphological similarity might be the most accurate and robust method to reflect information transfer between brain regions (Seidlitz et al., 2018; Li et al., 2021), and different spatial resolutions of measuring morphological similarity could detect spatial robustness of human brain structural network underlying state and trait anxiety. This was the first study to examine spatial robustness of human brain networks and characterized the dependence of topological mechanisms of anxiety on spatial resolution. The above temporal and spatial robustness studies would enhance our understanding of temporal and spatial organization of human brain networks. In summary, our study systematically examined the topological mechanisms of state and trait anxiety from both structural and functional networks perspectives, as well as from their spatial and temporal variations perspectives. Our results indicated that state and trait anxiety shared common brain network topology mechanisms, and the common topological mechanisms exhibited high temporospatial robustness.

In conclusion, we explored topological properties underlying state and trait anxiety based on human brain structural and functional network, as well as their spatial and temporal variations. We found state and trait anxiety were both positively correlated with pagerank centrality in the right insula, median and mode of pagerank, and degree centrality in the right insula and negatively correlated with CV of nodal efficiency in the left frontal eyes field of volume network. Our results demonstrated that state and trait anxiety shared common brain network topological mechanisms with high temporospatial robustness and would enhance our understanding of spatial and temporal organization of human brain.

### Limitations

Several limitations should be taken into consideration for this study. First, our sample size was small but across a large age span, and we need to increase the number of participants in future studies. Second, our study only analyzed human brain information of cerebral cortex but did not involve subcortical data. Anxiety as a negative emotional state, subcortical information such as amygdala, hippocampus, and caudate should be considered in future explorations. Finally, we only conducted inter-regional correlations and intra-regional vertex-wise analysis, and whole-brain vertex-wise analysis required immense memory storage or efficient computational capacity.

## Data Availability Statement

The raw data supporting the conclusions of this article will be made available upon request to the corresponding author.

## Ethics Statement

The studies involving human participants were reviewed and approved by the Institutional Review Board of Institute of Psychology Chinese Academy of Sciences. The patients/participants provided their written informed consent to participate in this study.

## Author Contributions

LJ: conceptualization and methodology. YL and LJ: formal analysis, investigation, and writing and editing. All authors contributed to the article and approved the submitted version.

## Funding

This study was supported by the National Natural Science Foundation of China (11674388).

## Conflict of Interest

The authors declare that the research was conducted in the absence of any commercial or financial relationships that could be construed as a potential conflict of interest.

## Publisher's Note

All claims expressed in this article are solely those of the authors and do not necessarily represent those of their affiliated organizations, or those of the publisher, the editors and the reviewers. Any product that may be evaluated in this article, or claim that may be made by its manufacturer, is not guaranteed or endorsed by the publisher.
