# Supplementary material for: State and Trait Anxiety Share Common Network Topological Mechanisms of Human Brain
Source: Front Neuroinform. 2022 Jun 23;16:859309. doi: 10.3389/fninf.2022.859309 (PMC9260038; doi:10.3389/fninf.2022.859309)
Supplement: Supplementary file 1 [file Data_Sheet_1.docx]

**Table S1. Vertex-wise functional connectivity network also demonstrated common topological mechanisms of state and trait anxiety.**

| Topological measures | Behavior | Vertex id | Region | *r* | *Corrected p* |
| --- | --- | --- | --- | --- | --- |
| Degree centrality | State anxiety | 1645 | LH_SomMot | 0.5505 | 0.0431 |
|  | State anxiety | 2182 | LH_SomMot | 0.5909 | 0.0132 |
|  | Trait anxiety | 9260 | RH_Vis | 0.5695 | 0.0246 |
|  | State anxiety | 10422 | RH_SalVentAttn_FrOper | 0.5702 | 0.0238 |
|  | Trait anxiety | 10422 | RH_SalVentAttn_FrOper | 0.5750 | 0.0246 |
|  | State anxiety | 12515 | RH_Default_PFCm | 0.6083 | 0.0109 |
|  | Trait anxiety | 12515 | RH_Default_PFCm | 0.5768 | 0.0246 |
| Eigenvector centrality | State anxiety | 11607 | RH_Cont_PFCl | 0.6244 | 0.0045 |
| PageRank centrality | State anxiety | 2182 | LH_SomMot | 0.5972 | 0.0192 |
|  | Trait anxiety | 9260 | RH_DorsAttn_Post | 0.5796 | 0.0459 |

**Table S2. The detailed information about groups with different levels of anxiety.**

| **Groups** | **Dimension** | **Average (Range)** | **Participants** |
| --- | --- | --- | --- |
| High state - high trait | State anxiety | 43.62 (38-66) | 21 |
|  | Trait anxiety | 44.14 (38-57) | 21 |
| High state - low trait | State anxiety | 39.00 (39-39) | 3 |
|  | Trait anxiety | 35.33 (35-36) | 3 |
| Low state - high trait | State anxiety | 34.40 (32-36) | 5 |
|  | Trait anxiety | 40.40 (38-43) | 5 |
| Low state - low trait | State anxiety | 27.32 (20-36) | 31 |
|  | Trait anxiety | 27.19 (20-36) | 31 |

*Note. state, state anxiety; trait, trait anxiety.*

**Table S3. The detailed information about groups with different levels of state anxiety.**

| **Groups** | **Average (Range)** | **Participants** |
| --- | --- | --- |
| High state anxiety | 43.04 (38-66) | 24 |
| Low state anxiety | 28.31 (20-36) | 36 |

**Table S4. Brain regions exhibiting significant topological differences between high state anxiety group and low state anxiety group.**

| **Network** | **Brain regions** | **Topology** | ***F*** | ***Corrected p*** |
| --- | --- | --- | --- | --- |
| Area (sm6) | LH_DorsAttn_FEF | Local efficiency | 13.10 | 0.0167 |
| Area (sm6) | - | Global efficiency | 6.07 | 0.0187 |
| Area (sm6) | LH_DorsAttn_FEF | CV of betweenness | 9.71 | 0.0433 |
| Area (sm6) | RH_SalVentAttn_Med | CV of betweenness | 10.62 | 0.0433 |
| Function (sm0) | RH_SalVentAttn_FrOper | Pagerank | 10.93 | 0.0489 |
| Function (sm0) | RH_SalVentAttn_FrOper | Median of degree | 11.39 | 0.0397 |
| Function (sm0) | LH_SalVentAttn_FrOper | Median of eigenvector | 11.97 | 0.0306 |

*Note. CV, Coefficient of variation；*

**Table S5. The detailed information about groups with different levels of trait anxiety.**

| **Groups** | **Average (Range)** | **Participants** |
| --- | --- | --- |
| High state anxiety | 43.42 (38-57) | 26 |
| Low state anxiety | 27.91 (20-36) | 34 |

**Table S6. Brain regions exhibiting significant topological differences between high trait anxiety group and low anxiety group.**

| **Network** | **Brain regions** | **Topology** | ***F*** | ***Corrected p*** |
| --- | --- | --- | --- | --- |
| Area (sm6) | LH_DorsAttn_FEF | CV of betweenness | 13.08 | 0.0461 |
| Area (sm6) | - | Global efficiency | 4.77 | 0.0331 |
| Function (sm0) | LH_DorsAttn_FEF | Degree centrality | 10.02 | 0.0371 |
| Function (sm0) | RH_DorsAttn_PrCv | Degree centrality | 12.67 | 0.0225 |
| Function (sm0) | - | Global efficiency | 5.22 | 0.0260 |
| Function (sm0) | LH_Vis | Local efficiency | 9.63 | 0.0443 |
| Function (sm0) | RH_DorsAttn_Post | Local efficiency | 15.90 | 0.0057 |
| Function (sm0) | LH_Limbic_TempPole | FWHM of Pagerank | 11.95 | 0.0308 |

*Note, FWHM, Full Width at Half Maximum; CV, Coefficient of variation；*

**Table S7. The detailed information about groups with different levels of total score of anxiety.**

| **Groups** | **Dimension** | **Average (Range)** | **Participants** |
| --- | --- | --- | --- |
| High anxiety | State anxiety | 43.62 (38-66) | 21 |
|  | Trait anxiety | 44.14 (38-57) | 21 |
| Low anxiety | State anxiety | 27.32 (20-36) | 31 |
|  | Trait anxiety | 27.19 (20-36) | 31 |

**Table S8. Brain regions exhibiting significant topological differences between high anxiety group and low anxiety group.**

| **Network** | **Brain regions** | **Topology** | ***F*** | ***Corrected p*** |
| --- | --- | --- | --- | --- |
| Area (sm6) | RH_SalVentAttn_TempOccPar | Degree centrality | 11.85 | 0.0316 |
| Area (sm6) | - | Global efficiency | 6.19 | 0.0353 |
| Area (sm6) | LH_DorsAttn_FEF | CV of betweenness | 12.16 | 0.0361 |
| Function (sm0) | RH_DorsAttn_PrCv | Degree centrality | 12.88 | 0.0226 |
| Function (sm0) | - | Global efficiency | 4.42 | 0.0405 |
| Function (sm0) | LH_Limbic_TempPole | FWHM of pagerank | 12.30 | 0.0290 |
| Function (sm0) | LH_SalVentAttn_FrOper | Median of eigenvector | 11.89 | 0.0346 |

*Note, FWHM, Full Width at Half Maximum; CV, Coefficient of variation；*

**Figure S1.** The detailed topological properties for different spatial scales and different time slots of brain regions with significant anxiety correlations. The upper part of the figure showed pagerank centrality of the RH_SalVentAttn_FrOper for different time windows, the middle part of the figure showed nodal efficiency of the LH_DorsAttn_FEF for different spatial scales and the lower part of the figure showed degree centrality of the LH_Default_PFC for different spatial scales.

**Figure S2.** Partial correlations between temporal variations of topological measures of human brain functional networks and anxiety: trait anxiety was positively associated with median (r = 0.3991, corrected p = 0.0381) and mode (r = 0.4265, corrected p = 0.0325) of pagerank centrality in the LH_SalVentAttn_FrOper, median (r = 0.4104, corrected p = 0.0381) and FWHM (r = 0.4242, corrected p = 0.0360) of betweenness centrality in the LH_SalVentAttn_FrOper, and mode (r = 0.4909, corrected p = 0.0325) of pagerank centrality in the RH_SalVentAttn_FrOper.

**Figure S3.** Partial correlations between temporal variations of topological measures of human brain functional networks and anxiety: both state and trait anxiety were positively correlated with median of betweenness centrality (state anxiety: r = 0.3991, corrected p = 0.0381; trait anxiety: r = 0.4322, corrected p = 0.0295) and mode of degree centrality (state anxiety: r = 0.4289, corrected p = 0.0326; trait anxiety: r = 0.4308, corrected p = 0.0308) in the RH_SalVentAttn_FrOper.
