# Supplementary figures and images for: State and Trait Anxiety Share Common Network Topological Mechanisms of Human Brain
Source: Front Neuroinform. 2022 Jun 23;16:859309. doi: 10.3389/fninf.2022.859309 (PMC9260038; doi:10.3389/fninf.2022.859309)

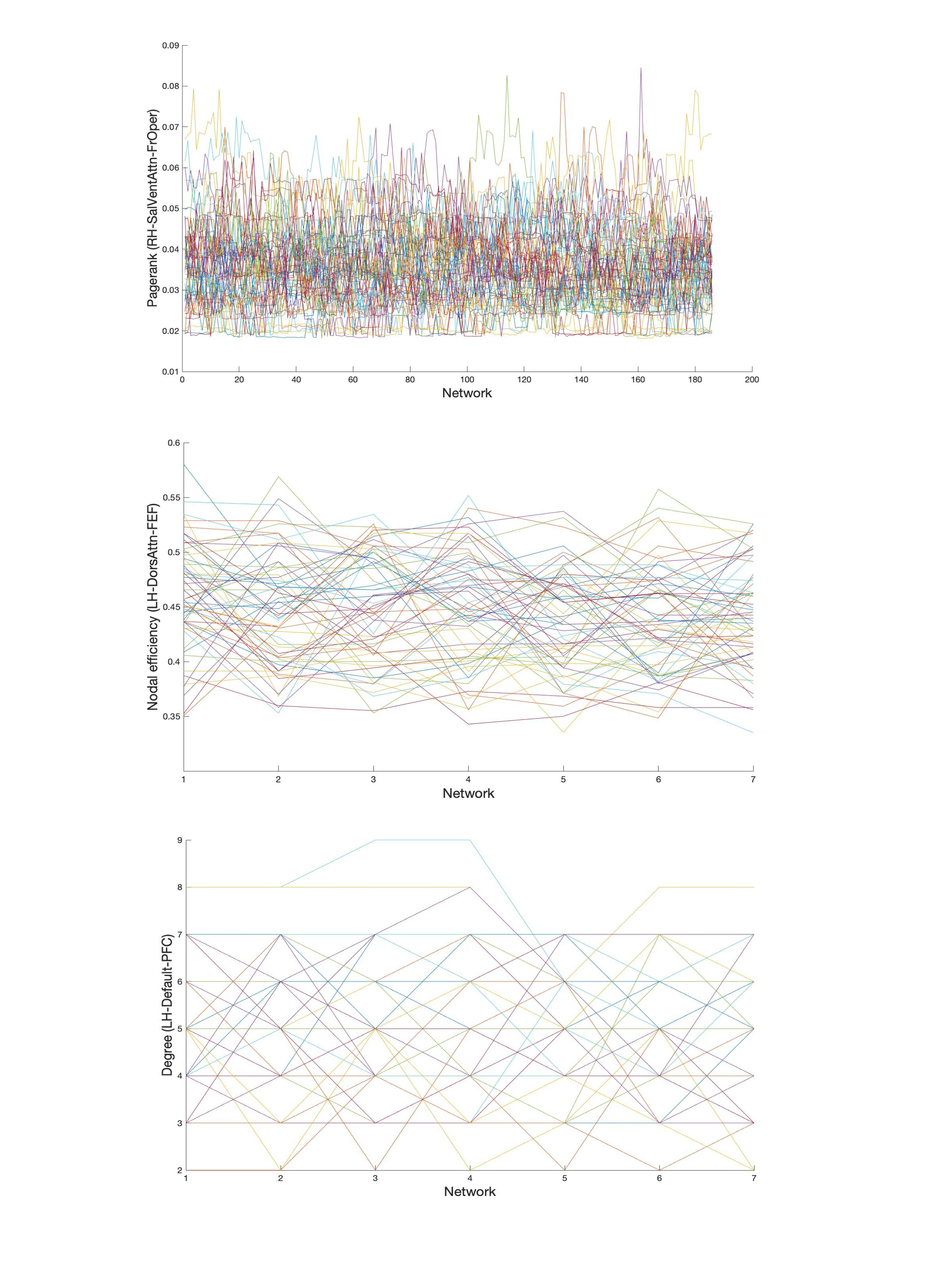

Supplement: Supplementary file 2 [file Image_1.TIF]

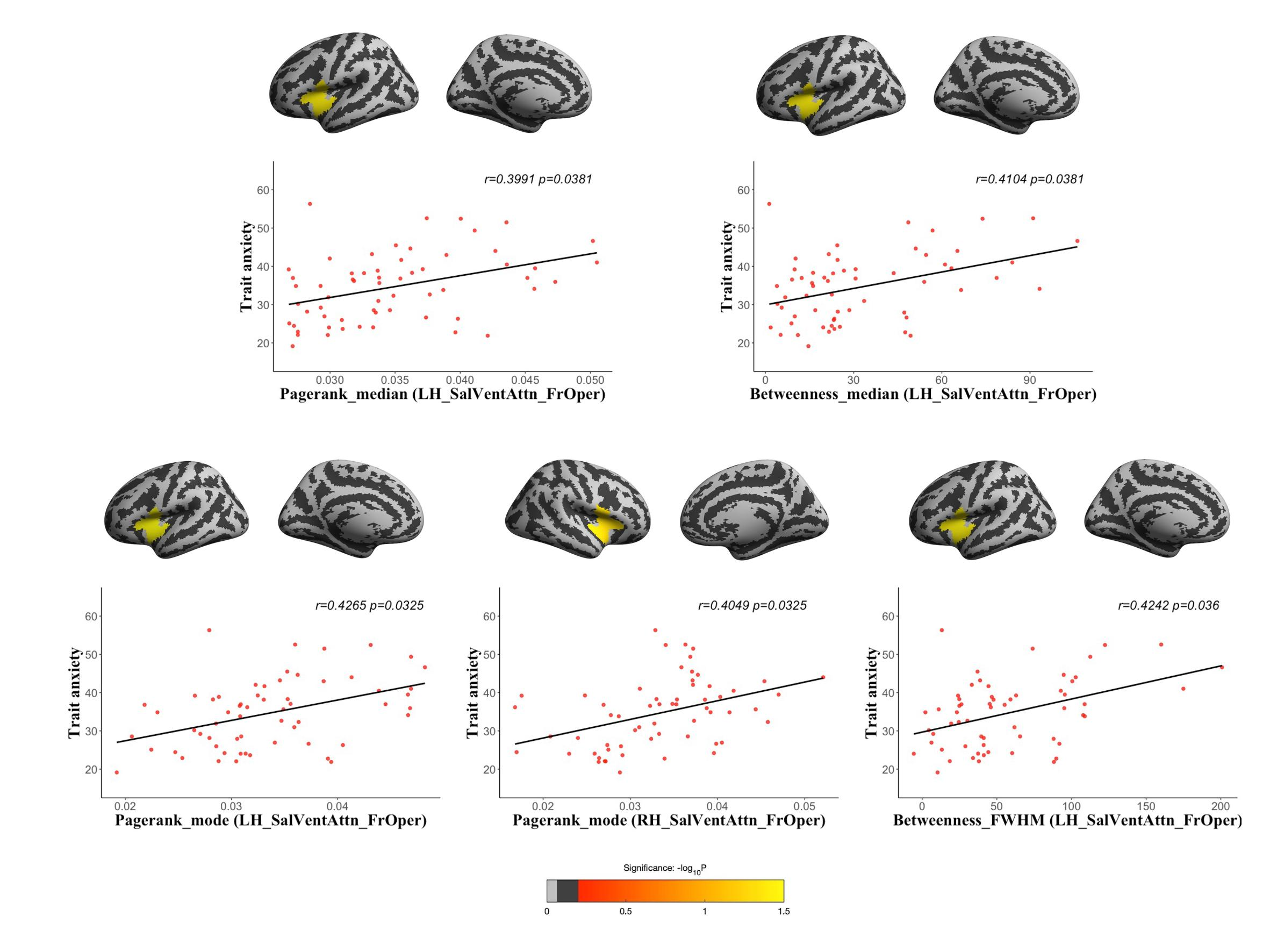

Supplement: Supplementary file 3 [file Image_2.TIF]

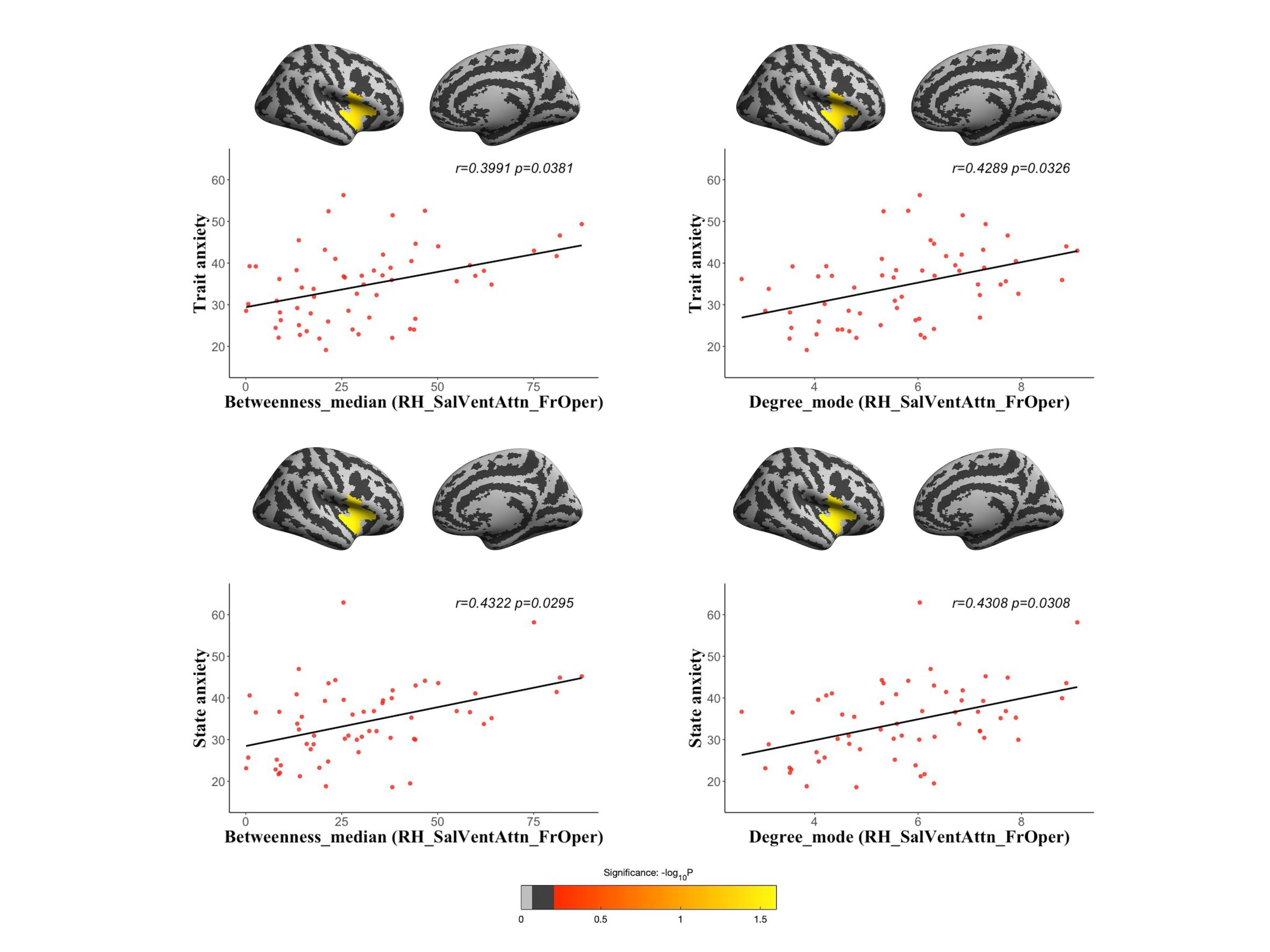

Supplement: Supplementary file 4 [file Image_3.TIF]
